# Influence of Embedding Fiber Optical Sensors in CFRP Film Adhesive Joints on Bond Strength

**DOI:** 10.3390/s20061665

**Published:** 2020-03-17

**Authors:** Neele Grundmann, Hauke Brüning, Konstantinos Tserpes, Tim Strohbach, Bernd Mayer

**Affiliations:** 1 Fraunhofer Institute for Manufacturing Technology and Advanced Materials IFAM, Wiener Straße 12, 28359 Bremen, Germany; 2 Laboratory of Technology & Strength of Materials, Department of Mechanical Engineering & Aeronautics, University of Patras, Patras 26500, Greece; 3 University of Bremen, Faculty of Production Engineering, 28359 Bremen, Germany

**Keywords:** structural health monitoring (SHM), sensor integration, fiber Bragg grating (FBG), carbon fiber reinforced plastic (CFRP), quasi-static tensile shear strength, tensile shear fatigue strength, epoxy-based film adhesive, adhesive layer thickness, fiber diameter, fibre coating

## Abstract

The increasing utilization of carbon fiber reinforced plastic (CFRP) in the aeronautical industry calls for a structural health monitoring (SHM) system for adhesively bonded CFRP joints. Optical glass fiber with inscribed fiber Bragg gratings (FBGs) is a promising technology for a SHM system. This paper investigates the intrusive effect of embedding optical glass fibers carrying FBGs on adhesive bond strength and adhesive layer thickness and quality. Embedding the optical glass fibers directly in the adhesive bond has the advantage of directly monitoring the targeted structure but poses the risk of significantly reducing the bond strength. Optical glass fibers with different cladding diameters (50, 80, 125 µm) and coating types (polyimide, with a thickness of 3−8 µm, and acrylate, with a thickness of ~35 µm) are embedded in structural and repair film adhesives here. Without embedded optical glass fibers, the film adhesives have an adhesive layer thickness of ~90 µm (structural) and ~100 µm (repair) after curing. The intrusive effect of the fiber embedding on the adhesive bond strength is investigated here with quasi static and fatigue single lap joint (SLJ) tensile shear tests. Also, the influence of hydrothermal aging procedures on the quasi static tensile shear strength is investigated. It is found that optical glass fibers with a total diameter (glass fiber cladding + coating) of ~145 µm significantly reduce the quasi static tensile shear strength and increase the adhesive layer thickness and number of air inclusions (or pores) in the structural film adhesive joints. In the repair adhesive joints, no significant reduction of quasi static tensile shear strength is caused by the embedding of any of the tested fiber types and diameters. However, an increase in the adhesive layer thickness is detected. In both adhesive films, no effect on the quasi-static tensile shear strength is detected when embedding optical glass fibers with total diameters <100 µm. The applied aging regime only affects the repair film adhesive joints, and the structural film adhesive joints show no significant reduction. A polyimide-coated 80 µm optical glass fiber is selected for fatigue SLJ tensile shear tests in combination with the more sensitive structural film adhesive. No significant differences between the S-N curves and tensile shear fatigue strength of the reference samples without embedded optical fibers and the samples carrying the polyimide-coated 80 µm optical glass fibers are detected. Thus, it is concluded that the influences of embedding optical glass fibers with total diameters <100 µm on the fatigue limit of the tested film adhesive joints is negligible.

## 1. Introduction

The utilization of carbon fiber reinforced plastic (CFRP) in the aircraft construction industry has continuously increased in the past decade [1]. Adhesive bonding is better suited for joining CFRP materials than conventional techniques like riveting or bolting [2,3]. However, the aeronautical industry sets high security standards. Without a method for identifying the condition of an adhesive bond, airworthiness authorities will not approve adhesive bonding for primary aircraft structures [3]. A structural health monitoring (SHM) system for monitoring and identifying the condition and remaining life of an adhesive bond would be a strong supporting factor for efforts to obtain airworthiness approval. Due to their small dimensions and characteristics, optical glass fibers carrying fiber Bragg gratings (FBGs) are suitable for the structural health monitoring of composite adhesive bonds [4]. 

**Figure 1:**
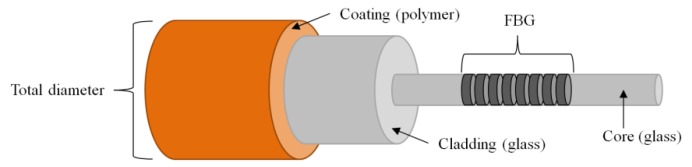
Schematic sketch of the structure of an optical glass fiber with a fiber Bragg grating (FBG) (redrawn and changed after a sketch from [6] with permission from Yang and the *ETZ* journal, published by VDE Verlag GmbH, 2010).

An optical glass fiber consists of a fragile glass core and cladding protected by a surrounding polymer coating (see Figure 1) [5]. The FBG sensor is a periodic variation of the effective refractive index and only makes up a small portion of the entire glass fiber core. When the optical glass fiber is elongated or bent in the area of the FBG, it senses the strain changes [5].

In earlier studies [7,8,9], optical fibers with inscribed FBGs have been embedded in the adherents. Standard telecommunication optical glass fibers have been employed in this context. With this set-up, researchers have successfully monitored disbond initiation and crack growth in the adhesive [7,8,9,10]. In 2014, three studies were published [4,11,12] that placed the optical glass fiber directly in the adhesive bond. This set-up has the advantage of directly monitoring the stress distribution in the adhesive [11]. However, placing the optical fiber directly in the adhesive creates the risk of significantly reducing the bond strength [4,12]. The intrusive impact of the embedded optical fiber on the adhesive bond should be minimal. In the past, only little attention has been paid to the impact that placing a fiber sensor in the adhesive has on the bond strength and adhesive layer thickness. Previous investigations were carried out by da Silva et al. in 2014 [4] and Canal et al. in 2014 [12], using single lap (SL) tensile shear tests. Canal et al. [12] compared the impact of embedding multiplexed FBG arrays (acrylate-coated 125 µm glass fibers) in three different positions in the two-component epoxy adhesive Araldite 420 A/B, namely, between the 1st and 2nd CFRP ply, in the adherent-adhesive interface, and in the adhesive. No negative impact on joint performance was discovered (n = 4). In 2014, da Silva et al. [4] investigated the effect of embedding optical fiber sensors (no specification) in the SikaForce^®^-7888 L10 adhesive (a steel adherent). They found that embedding optical fiber sensors directly in the adhesive can cause a significant reduction in the bond strength.

In the past, the research regarding embedding optical fibers in bonded joints for SHM purposes has mainly focused on the application of the system, i.e., for defect detection and monitoring. Various optical fibers with different diameters and coating types have been embedded in structural and non-structural film adhesives at various curing temperatures. Detailed attention has been paid to the effect that the embedding has on the sensor (e.g., [13,14,15,16]) but not on the effect that it has on the bonded joint strength and performance. Only very limited research [4,12] is available on this topic, although maintaining quasi-static and fatigue strength is a major issue for the aeronautical industry and airworthiness approval when embedding sensors in aircraft structures. Therefore, this study investigates the influence of embedding different optical glass fiber types on the quasi-static and fatigue tensile shear strength of bonded CFRP joints. Thus, the relevant gap in airworthiness certification related to knowledge about embedded fiber optical sensors for monitoring adhesive joints is addressed here. To gain a broader perspective and knowledge about the consequences of using embedded optical fibers, the adhesive layer thickness and quality are analyzed with different embedded optical fiber types and are related back to the bond strength and performance. Since structural and repair film adhesives are widely used in the aeronautical industry for bonding load bearing primary CFRP structures, a strong focus of this study is embedding fiber optical sensors in these adhesives. The embedding of fiber optical sensors in film adhesives has not been studied so far, although film adhesives present multiple unique challenges for embedding of fiber optical sensors, namely, the small adhesive layer thickness, high curing temperatures, and the fabric carrier. The findings of the material testing conducted here are related back to the influences on sensor function and performance.

## 2. Materials and Methods

### 2.1. Materials

The employed fibers were tested with commercially available acrylate and polyimide coatings. The properties of the standard optical glass fiber acrylate coating and the high-temperature specialty polyimide coating are presented in Table 1.

The employed optical glass fibers were manufactured by RISE Acreo Fiberlab (Sweden) and had cladding diameters of 50, 80, and 125 µm. The protective acrylate or polyimide coating was applied directly after cooling. Depending on the glass fiber diameter, the acrylate coating was applied with a thickness of 35–40 µm and a thickness of 3–8 µm for the polyimide coating. The total fiber diameters resulting from the combination of different cladding diameters and coating types are presented in Table 2. In this study, the fiber types are named as specified in Table 2.

The employed adhesives were the industrially available structural film adhesive FM^®^ 300K and the repair film adhesive FM^®^ 300-2K, manufactured by Cytec Industries Inc. (USA). Both were distributed on a polyester fabric carrier [20,21]. The material properties of these film adhesives are stated in the data sheets provided by the manufacturer [20,21]. The film adhesives were processed and cured according to the manufacturer’s specifications at 121 °C (FM^®^ 300-2K) and 175 °C (FM^®^ 300K), respectively. After curing, the film adhesives have an adhesive layer thickness of ~90 µm (FM^®^ 300K) and ~100 µm (FM^®^ 300-2K) in a bond with CFRP adhesives, respectively.

### 2.2. Quasi-Static Tensile Shear Tests

**Figure 2:**
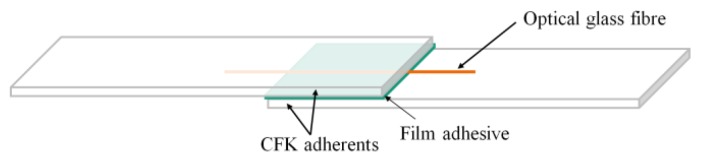
Sketch of the lay-up of a single lab shear specimen with one optical glass fiber embedded in the adhesive.

The CFRP single lap (SL) adhesive joints for tensile shear testing were prepared according to the DIN EN 1465:2009-07 standard [22]. The CFRP adherents were cut from a HexPly^®^ 8552/33%/UD134/IM7 CFRP sheet with a (45/−45/0/90/0/90/−45/+45/+45/−45/0/90) s lay-up and 2.8 mm sheet thickness. For specimen preparation, the adherents were sandpapered by hand and cleansed with 2-propanol. One layer of film adhesive (25 × 12.5 mm) was placed on the lower adherent and the glass fiber was placed along the central line on the film adhesive before placing the upper adherent on top and applying pressure (see Figure 2). Specimens for all fiber diameter/coating/adhesive combinations were manufactured (n = 12). In addition, reference specimens without an embedded glass fiber were prepared (n = 12). A jig was used for the precise alignment of the two adherents. The curing took place in a hot-air autoclave by Scholz Maschinenbau (Germany) according to the adhesive manufacturer’s specifications.

**Figure 3:**
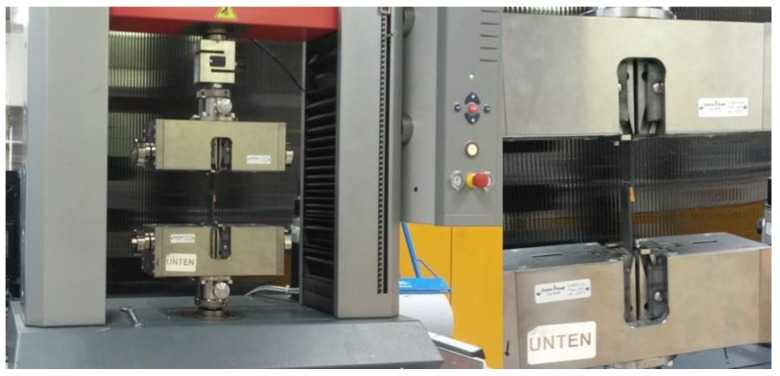
Test set-up for the quasi-static tensile shear test with a single lab shear FM^®^ 300-2K specimen clamped in a Z020 ZwickRoell universal testing machine with a 20 kN load cell at Fraunhofer IFAM (Bremen, Germany).

To simulate aging, half of the specimens from each sample were placed in 70 °C deionized water for 1000 h. After 1000 h, the specimens were removed from the water and dried at room climate and temperature for two hours to prevent water dripping from the specimens during testing. The specimens were tested after two hours of drying. The quasi-static tensile shear tests were performed with a ZwickRoell Z020 universal testing machine and a 20 kN load cell (see Figure 3) according to the DIN EN 1465:2009-07 standard [22]. A testing speed of 10 mm/min was applied. The tests were controlled via a computer running ZwickRoell testXpert^®^ II software and a ZwickRoell controller. The tests were performed in standard laboratory conditions. After finishing the tests, the fracture surfaces of the specimen were visually examined and evaluated. The bonded area for the calculation of the tensile shear strength was corrected according to the findings of the fracture surface examination.

### 2.3. Measuring of Adhesive Layer Thickness in Cross-Sectional CFRP Bond Cuts 

**Figure 4:**
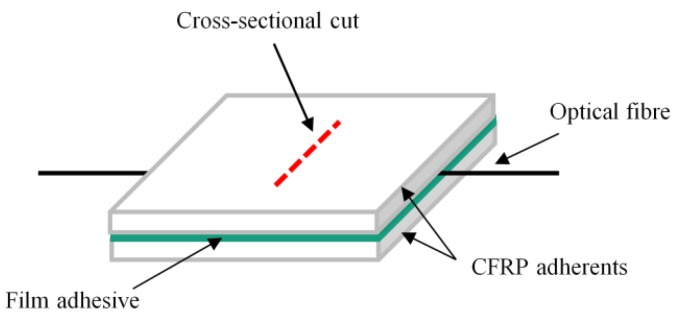
Schematic sketch of the lay-up of a bonded joint specimen with an optical glass fiber embedded in the adhesive. The red dashed line indicates the position where the cross-sectional cuts for measuring the adhesive layer thickness taken from.

The specimens were manufactured by bonding two CFRP slates (100 × 100 × 2 mm) with the FM^®^ 300-2K and FM^®^ 300K film adhesives and embedding one glass fiber per specimen, as described in Section 2.2, demonstrated in Figure 4. The specimens were manufactured for all glass fiber diameter and coating combinations. Additionally, reference specimens without an embedded glass fiber were manufactured. Deviating from the description in Section 2.2, the curing of the specimens took place under vacuum bagging. The cross-sectional cuts were cut from the position indicated by the red dashed line in Figure 4 with a diamond cutting disc and then embedded in the two-component epoxy resin EpoFix by Struers GmbH (Hannover, Germany). The embedded cross-sectional cuts were first grinded with grit sizes ranging from 80 to 4000 and then polished in the following steps: (1) Buff MD-Largo with a 3 µm suspension and lubricant (300 U/min, 25 N, 3 min); (2) polishing cloth MM414 with BioDiamant Liquid MM 140, 1 µm (300 U/min, 35 N, 5 min); (3) polishing cloth MD-Chem with an OP-S suspension, 0.25 µm (150 U/min, 25 N, 2.5 min). Photos of the cross-sectional cuts were taken with a DMRX light microscope by Leica Microsystems (Wetzlar, Germany) and the Image Access standard software at a 200 × magnification. The adhesive layer thickness was measured by the Image Access standard software.

### 2.4. Air Inclusions in the Adhesive Layer

The specimens were manufactured as described in 2.2. Deviating from that description, transparent glass slates (100 × 100 × 2 mm) were used as adherents. The adherents were only cleansed with 2-propanol and not sandpapered. Curing did not take place under vacuum bagging conditions, but pressure plates were placed on each specimen to achieve good results. Photos were taken with a Canon EOS40D camera and an EF 50 mm/2.5 macro lens manufactured Canon (Tokyo, Japan) and cropped with the MATLAB Image Viewer. A MATLAB-based image recognition and analysis algorithm was used to analyze the percentage of air inclusions (or pores) in each sample. 

### 2.5. Tensile Shear Fatigue Tests

**Figure 5:**
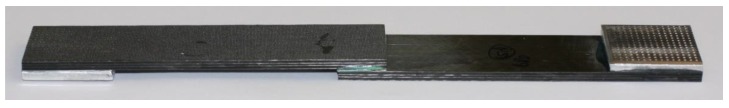
FM^®^ 300K single lab shear specimen with aluminium tabs fixed on both ends of the specimen.

Deviating from the description in Section 2.2, the CFRP adherents were cut from a CFRP sheet with a (0/90/+45/−45/0/90) s lay-up. The CFRP sheet was manufactured from an epoxy-based prepreg with 34% matrix mass (M21) and 268 g/m² fiber mass (IMA-12K by Hexcel, Stamford, USA). The adherent dimensions and manufacturing procedure were equivalent to the quasi-static single lap shear specimens. Deviating from the description in Section 2.2., aluminium tabs were fixed on both ends of the specimen, as indicated in Figure 5.

**Figure 6:**
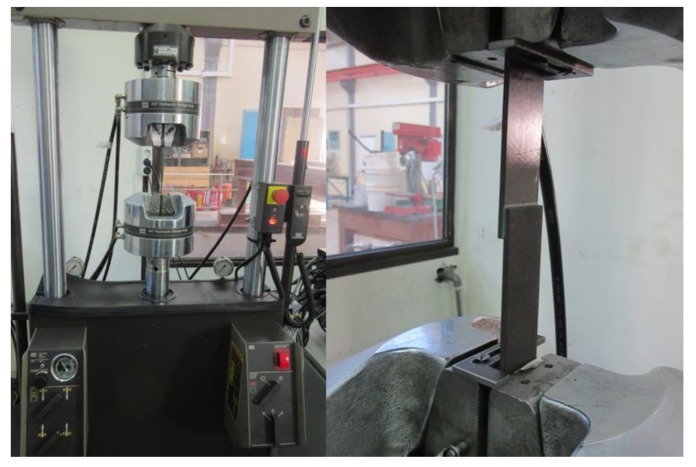
Test set-up for the tensile shear fatigue test with a single lab shear FM^®^ 300K specimen clamped in an MTS 810 servo hydraulic testing machine with a 100 kN load cell at the University of Patras (Greece).

Two samples were manufactured, namely, a reference sample with the FM^®^ 300K film adhesive, without an embedded optical glass fiber, and a sample with the FM^®^ 300K film adhesive, with one embedded polyimide-coated 80 µm optical glass fiber. For each sample, 20 specimens were manufactured and tested. Four tests were performed at five stress levels, namely, 60%, 53%, 43%, 37% and 35% of the quasi-static shear strength of the reference samples. The tensile shear fatigue tests were performed with a MTS 810 servo hydraulic testing machine (see Figure 6) and a 100 kN load cell according to the ISO 13003:2003-12 standard [23]. The tests were controlled via a computer running an Instron controller and the Instron Dynamic software WaveMatrix^TM^. A constant amplitude sinusoidal waveform at a frequency of 7 Hz was applied. The applied stress ration was R = F_min_/F_max_ = 0.1. The tests were carried out in the load control mode. The terminating condition was the fracturing of the tested specimen. The tests were performed in standard laboratory conditions. The evaluation of the test results was conducted according to the ISO 12107:2012-08 standard [24]. 

## 3. Results

### 3.1. Quasi-Static Tensile Shear Strength

**Figure 7:**
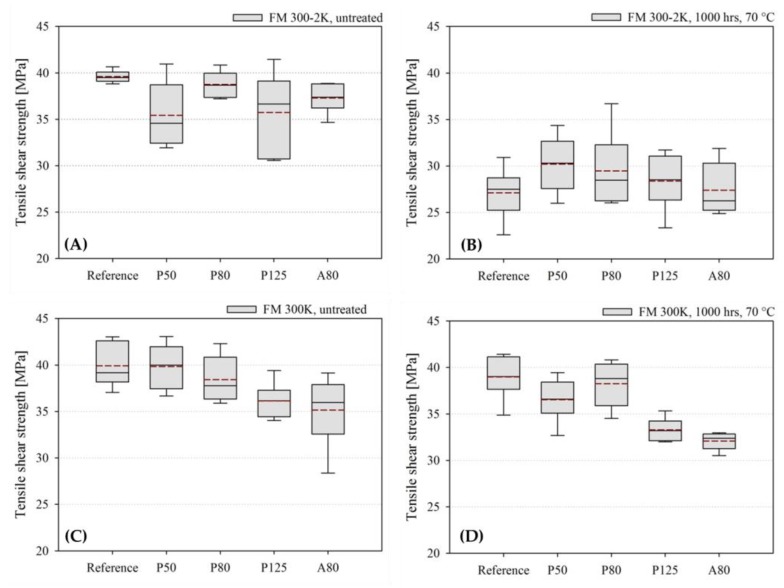
Boxplot diagrams of the quasi-static tensile shear strength values for the single lap FM^®^ 300-2K (**A**) and (**B**) and FM^®^ 300K (**C**) and (**D**) samples. (**A**) Quasi-static tensile shear strength of the untreated FM^®^ 300-2K samples (n = 6). (**B**) Quasi-static tensile shear strength of the aged FM^®^ 300-2K samples (water immersion, 70 °C, 1000 h, n = 6). (**C**) Quasi-static tensile shear strength of the untreated FM^®^ 300K samples (n = 6). (**D**) Quasi-static tensile shear strength of the aged FM^®^ 300K samples (water immersion, 70 °C, 1000 h) (n = 6). The median is indicated by the solid black line and the average tensile shear strength is indicated by the dashed red line.

The results from the single lap tensile shear test for the untreated and aged samples carrying the different fiber diameter and coating combinations are presented in Figure 7 and Table 3.

The untreated FM^®^ 300-2K samples (n = 6) show a similar average tensile shear strength between the reference sample and the samples with the different embedded glass fiber types (see Figure 7 and Table 3). The tensile shear strength in the untreated FM^®^ 300-2K samples ranges between 39.6 ± 0.6 MPa for the reference sample without the embedded glass fiber and 35.4 ± 3.5 MPa for the P50 sample. No tendency is detectable in the average tensile shear strength development of FM^®^ 300-2K samples when embedding different glass fiber diameter and coating combinations. The applied aging procedure has caused a significant reduction in the average tensile shear strength of all FM^®^ 300-2K samples (see Figure 7B). The average tensile shear strength in the aged FM^®^ 300-2K samples ranges between 30.2 ± 3.0 MPa for the P50 sample and 27.1 ± 2.7 MPa for the reference sample. Again, no tendency is detectable. The examination of the fracture interfaces after testing revealed no abnormalities. The fracture was always interlaminar in the first or second layer of the CFRP of one or both adherents. The embedding of the P125 fibers increases the number of specimens in which the fracture reaches the second CFRP layer (67%) in comparison to the specimens carrying the other fiber types (0% to 50%). After aging, the number of P125 specimens in which the fracture reaches the second CFRP layer increases to 83%.

The untreated FM^®^ 300K samples (n = 6) carrying P50 and P80 optical glass fibers show an average tensile shear strength that is not significantly different to that of the reference sample (compare Figure 7 and Table 3). The applied aging procedure causes only a slight reduction for the reference sample and the P50 and P80 samples (see Figure 7 and Table 3). The untreated FM^®^ 300K samples carrying the P125 and A80 fibers show statistically significantly lower average tensile shear strengths than the reference sample. The applied aging regime causes a detectable reduction in the aged P125 and A80 samples in comparison to the untreated samples. In one untreated A80 specimen, two unaged P125 specimens, and one aged P125 specimen, the examination of the fracture interfaces reveals areas with the absence of a fully developed interface between the adhesive and the adherent. The results presented in Figure 7 and Table 3 have been corrected according to the effective bond area. Without corrections, according to the effective bonding area, the average tensile shear strength of the untreated P125 and A80 specimens would be approximately 50% lower. Furthermore, the examination of the fracture interfaces after testing revealed that the fracture was always interlaminar in the first, second, or third layer of the CFRP of one or both adherents. The embedding of the P125 and A80 fibers increases the number of specimens per sample in which the fracture reaches the third CFRP layer (50%) in comparison to the samples carrying the other fibers (33%). The applied aging procedure causes an increase in the number of P125 and A80 specimens per sample in which the fracture reaches the third CFRP layer (67% to 83%). In the other samples (e.g., the reference, P50, and P80) the number of specimens in which the fracture reaches the second CFRP layer increases from 33% before aging to 50% after aging.

**Figure 8:**
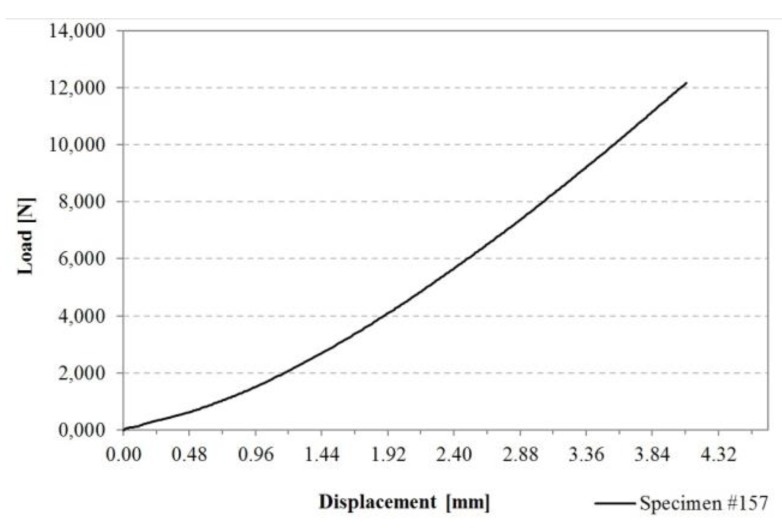
Exemplary load–displacement curve of an untreated FM^®^ 300K reference specimen (#157).

An exemplary load–displacement curve of an untreated FM^®^ 300K reference specimen is presented in Figure 8. The presented curve shows the brittle behaviour of the tested specimen. The specimen shows very little deformation during the tensile shear testing. 

The load–displacement curves of the other samples all show a shape and form similar to the one presented in Figure 8.

### 3.2. Adhesive Layer Thickness

**Figure 9:**
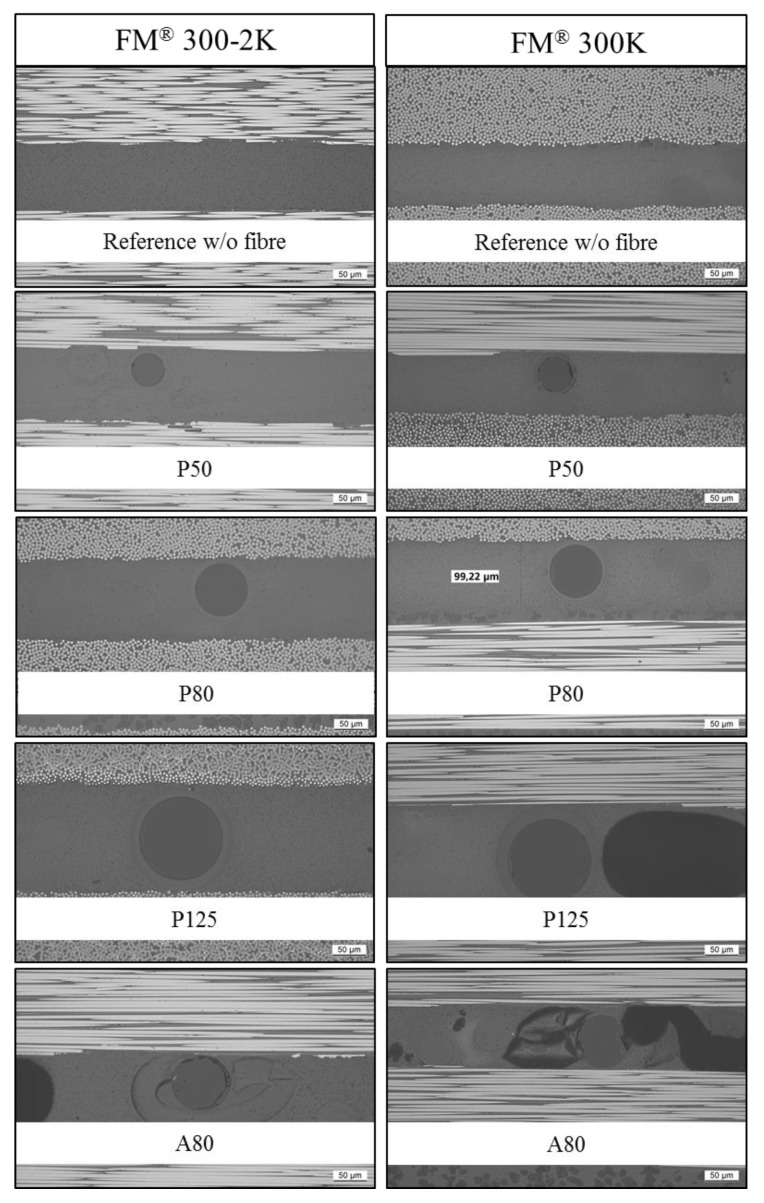
Photomicrographs of the cross-sectional cuts of adhesively bonded CFRP slates (200× magnification). The specimens in the left column were bonded with FM^®^ 300-2K and the specimens in right column were bonded with FM^®^ 300K. The specimens carry the following optical glass fiber types from top to bottom: No optical fiber, P50, P80, P125, and A80. In the FM^®^ 300K P125 specimen and both A80 specimens, air inclusions are visible in the adhesive layer (dark grey to black coloured areas).

Photomicrographs of the cross-sectional cuts are presented in Figure 9.

**Figure 10:**
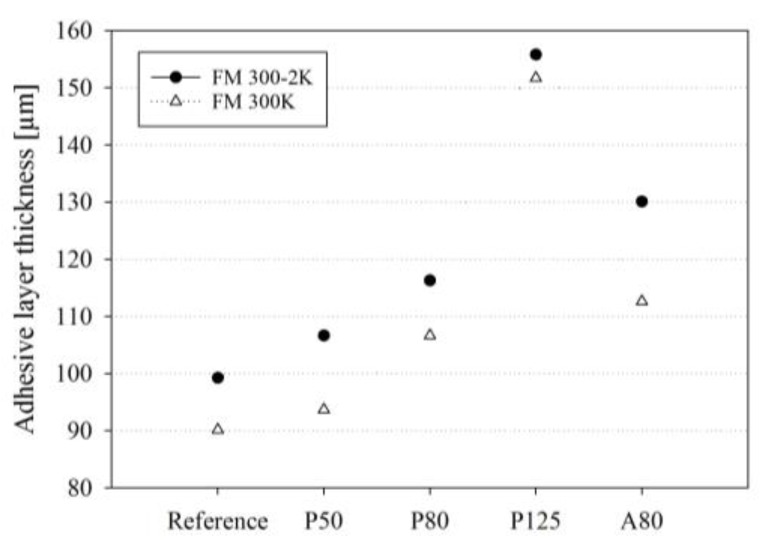
Changes in the adhesive layer thickness of FM^®^ 300K and FM^®^ 300-2K slate bonds caused by embedding optical fibers with different diameters and coating types (n = 1): Reference, P50, P80, P125, A80, and A125. The positions of the cross-sectional cuts are indicated in Figure 3.

In Figure 9, it is visible that the acrylate coating of the A80 fiber has separated from the optical glass fiber and is destroyed. Air inclusions (dark grey to black coloured areas) are visible in both A80 photomicrographs. Also, in the FM^®^ 300K specimen carrying the P125 fiber, an air inclusion (dark grey to black coloured areas) is visible to the right of the optical fiber. The P50 and P80 fibers seem to have sufficient space in the adhesive layer but are not positioned centrally in the bond line. The P125 and A80 fibers completely fill the bond line and only show a very thin adhesive layer between the fiber surface and the adherents. The adhesive layer thicknesses measured in the photomicrographs of the cross-sectional cuts are presented in Figure 10. For both adhesives, the measured adhesive layer thicknesses of the specimens carrying the P50 and P80 optical fibers are only slightly higher than that of the reference specimen (see Figure 10). The thickness peaked for specimens carrying the P125 fiber. In the specimens carrying the A80 optical fiber, the measured increase in adhesive layer thickness was slightly higher than that of the P80 fibers, but significantly lower than that of the specimens carrying the P125 optical glass fiber. In every case, the adhesive layer thickness of the FM^®^ 300-2K specimens was higher than that of the FM^®^ 300K specimens carrying the same glass fiber type (see Figure 10).

### 3.3. Air Inclusions in the Adhesive Layer

**Figure 11:**
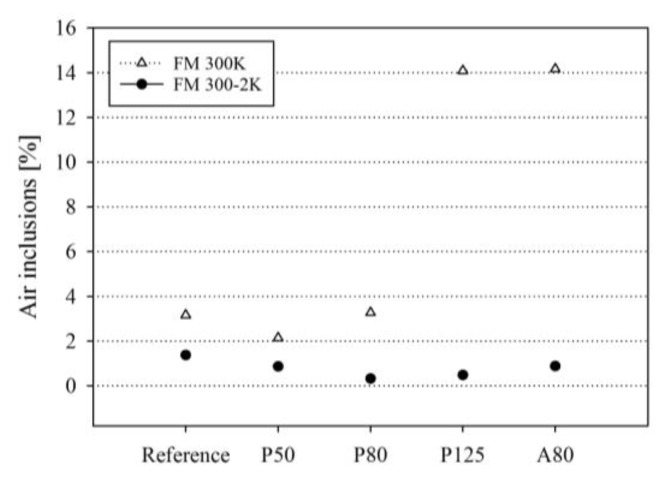
Air inclusions measured in the FM^®^ 300K and FM^®^ 300-2K glass slate bonds with different embedded optical glass fibers: Reference, P50, P80, P125, A80, and A125 (n = 1).

Figure 11 demonstrates that in all FM^®^ 300-2K specimens, the percentage of air inclusions is similar to that of the reference specimen. No increase or tendency is detectable here. In the case of the FM^®^ 300K adhesive, the percentage of air inclusions of the specimens carrying the P50 and P80 optical fibers is similar to that of the reference specimen. In the specimens carrying the P125 and A80 optical fibers, a significant increase in air inclusions is detectable (see Figure 11). In all FM^®^ 300K specimens, the percentage of air inclusions is higher than that in the FM^®^ 300-2K specimens.

### 3.4. Tensile Shear Fatigue Strength

**Figure 12:**
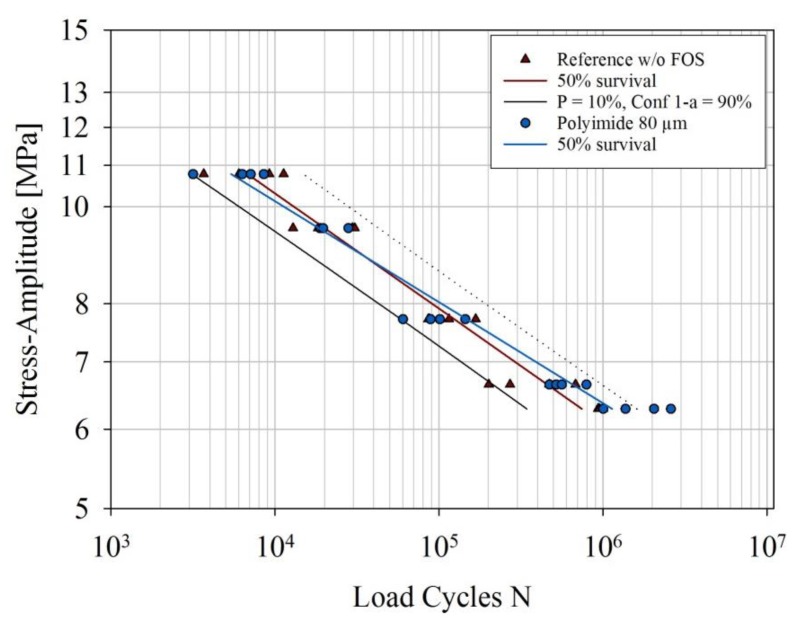
S–N curves of the two samples of specimens bonded with FM^®^ 300K. A reference sample without an embedded optical fiber is compared to a sample with an embedded P80 optical glass fiber. Per sample, four tests were performed at five stress levels, namely, 60%, 53%, 43%, 37%, and 35% of the quasi-static shear strength of the reference samples. The stress levels are plotted here against the logarithmic scale of the load cycles at failure. Here, R = F_min_/F_max_ = 0.1 and the applied frequency was 7 Hz.

The tensile shear fatigue strength of a FM^®^ 300K reference sample without an embedded optical fiber was compared to the tensile shear fatigue strength of a FM^®^ 300K sample with an embedded P80 optical glass fiber. A comparison of the S–N curves of the two FM^®^ 300K samples is presented in Figure 12.

The two S–N curves do not differ significantly. The P80 sample fails at a slightly lower number of load cycles at the highest stress level (10.77 MPa) in comparison to the reference sample. At the two lowest stress levels (6.64 MPa and 6.29 MPa), it fails at a slightly higher number of load cycles than the reference sample.

## 4. Discussion

The tensile shear strengths of the untreated reference samples are in accordance with the FM^®^ 300K and the FM^®^ 300-2K data sheets provided by the manufacturer [20,21]. Thus, the results of the tensile shear test are considered realistic. The results for the quasi-static tensile shear strength of the aged FM^®^ 300K reference samples are in accordance with the data sheet provided by the manufacturer [21]. With the applied aging regime, no significant influence on the quasi-static tensile shear strength of the FM^®^ 300K adhesive was detected. The corresponding data for the quasi-static tensile shear strength of aged FM^®^ 300-2K reference samples were not provided by the manufacturer. However, the detected reduction of the quasi-static tensile shear strength for the FM^®^ 300-2K samples (−15% to −32%) lies in the range described by Palmieri et al. in 2013 [25] for another epoxy-based structural film adhesive (AF-555M, −27%), although the aging period was much shorter in this study in comparison to the study by Palmieri et al. [25] (42 days vs. 360 days). For both adhesives, no influence of the different embedded optical glass fibers on the aging behaviour was detected. The load–displacement curves of all specimens are typical for single lap shear samples with a brittle behaviour, as shown for example by da Silva et al. in 2006 [26].

### 4.1. Influence of Embedding Optical Fibers with Total Diameters of <100 µm

Embedding optical glass fibers with total diameters <100 µm in the adhesive layer causes no significant reduction of the quasi-static tensile shear strength of both film adhesives. Additionally, no influence of embedding the exemplarily tested P80 optical fiber on the tensile shear fatigue strength of SLJ FM^®^ 300K bonded joints was detected. The fatigue limit of the fatigue specimen carrying the P80 optical fiber was even slightly higher at lower stress levels than that of the reference specimen. The embedding of the P50 and P80 optical fibers can therefore be considered successful. 

The observation that the quasi-static tensile shear strength is not affected by embedding optical fibers with total diameters <100 µm coincides with the finding that the fiber presence does not increase the percentage of air inclusions in the FM^®^ 300-2K adhesive layer. In the FM^®^ 300K adhesive, the detected increase of air inclusions is small. The embedding of the P50 and P80 optical fibers increased the adhesive layer thickness of the host specimen slightly in comparison to the reference samples (see Figure 10). However, it is concluded that in the case of the samples with embedded optical glass fibers with total diameters <100 µm, the increase is small enough to not cause a reduction of the quasi-static tensile shear strength.

### 4.2. Influence of Optical Fibers with Total Diameters of ~145 µm

#### 4.2.1. FM^®^ 300-2K

For the FM^®^ 300-2K adhesive bonds, it can be concluded that embedding optical glass fibers with total diameters of ~145 µm has no significant influence on the quasi-static tensile shear strength. Detecting no reduction in the quasi-static tensile shear strength coincides with not detecting an increase in the percentage of air inclusions in the FM^®^ 300-2K adhesive bonds. The results suggest that the FM^®^ 300-2K adhesive bonds have a higher tolerance for embedding optical glass fibers than the FM^®^ 300K adhesive bonds. This might be due to the slightly higher adhesive layer thickness of the FM^®^ 300-2K adhesive in comparison to the FM^®^ 300K adhesive (see Figure 10). The significant increase in adhesive layer thickness detected in this paper when embedding optical glass fibers with total diameters ~145 µm does not cause a significant reduction of the quasi-static tensile shear strength of FM^®^ 300-2K joints. In the FM^®^ 300-2K specimens, the results indicate that the separation of the acrylate coating does not affect the quasi-static tensile shear strength of the adhesive bond. The lower curing temperature of the FM^®^ 300-2K in comparison to the FM^®^ 300K might be an influencing factor.

#### 4.2.2. FM^®^ 300K

For FM^®^ 300K adhesive bonds, the results indicate that embedding optical glass fibers with total diameters of ~145 µm causes a significant reduction in quasi-static tensile shear strength in comparison to the reference sample. This finding coincides with the findings from other investigations presented in this study:

- Adhesive layer thickness: It was detected in this study that the embedding of glass fibers with total diameters of ~145 µm in FM^®^ 300K adhesive significantly increases the adhesive layer thickness. The FM^®^ 300-2K specimens with the same optical fibers show a comparable increase in adhesive layer thickness but no decrease in average quasi-static tensile shear strength. The detected smaller adhesive layer thickness in the A80 samples, in comparison to the P125 samples, is most likely related to the destruction of the acrylate coating in the curing process visible in Figure 9. However, in the FM^®^ 300-2K samples, the destruction of the acrylate coating was also detected (see Figure 9), but, in this group, it does not significantly influence the quasi-static tensile shear strength.
- Air inclusions: It was detected that the FM^®^ 300K specimens with A80 and P125 optical fibers show an increased percentage of air inclusions. This finding is likely related to the optical fiber diameters that cause a slightly uneven placement of the upper adherent, allowing air to be entrapped in the bonded joint. The finding of an increased number of air inclusions in the A80 and P125 samples coincides with the decrease in quasi-static tensile shear strength in the FM^®^ 300K samples carrying the A80 and P125 optical fibers. A similar relation was found by Bascom and Cottington in 1972 [27], where an increase of bond strength was achieved by improving manufacturing conditions. This manifests in a reduction of air inclusions in the adhesive as an indicator and is correlated to the increase in tensile shear strength by the authors.
- Non-fully developed interfaces: The observation of non-fully developed interfaces detected in one A80 and three P125 carrying specimens indicates that optical glass fibers with total diameters of 140–145 µm are too thick for embedding in FM^®^ 300K adhesive bonds. This finding supports the assumption from above, i.e., that the upper adherent might be placed slightly unevenly when an A80 or P125 optical glass fiber is embedded in the bond line. Furthermore, the fact that the uncorrected tensile shear strength is approximately 50% lower than the corrected one (the tensile shear strength has been corrected according to the actual effective bond area after discovering the non-fully developed interfaces, for further information, see Section 3.1.) in combination with the fact that in a real-life production environment the not-fully developed interfaces might not be discovered, presents a significant risk for the integrity of the host structure when it is in service. In the aeronautical industry this is not acceptable.
- Fracture depth: The finding that the fracture reaches the third CFRP layer in significantly more P125 and A80 specimens than in specimens with other embedded fibers indicates that the stress distribution in the adhesive and adherents is greatly influenced by the fiber diameter.

### 4.3. Key Aspects for Selecting an Appropriate Optical Glass Fiber for SHM Applications in FM^®^ 300-2K and FM^®^ 300K

Although the results indicate that the tested FM^®^ 300-2K repair film adhesive (with a layer thickness after curing of 100 µm) is more tolerant towards embedding different fiber diameters and coating types than the tested FM^®^ 300K adhesive film (layer thickness after curing of 90 µm), the same recommendations apply for both. It is concluded that:

- Acrylate coatings are not suitable for a SHM application in the tested structural and repair film adhesives due to the material properties of acrylate and the high curing temperatures of the tested film adhesives.
- The P50 and P80 optical glass fibers are suitable for SHM in the tested structural and repair film adhesives or similar adhesive types. Due to the fact that the handling of the P80 fiber is easier and less cost intensive than that of the P50 fibers, this study suggests the usage of P80 optical glass fibers.
- The finding that the embedding of P80 optical glass fibers does not influence the fatigue strength significantly is a promising result for the future utilization of FBGs of this fiber type for SHM applications in aircraft.
- A relation between the increase of air inclusions, caused by the presence of optical glass fibers in the cured adhesive, and the reduction in quasi-static tensile shear strength was detected. No relation between the reduction in quasi-static tensile shear strength and increase in adhesive layer thickness or separation of the acrylate coating was detected.

### 4.4. How Do the Findings from the Material Testing Influence the FBG Performance and Functionality?

Three main findings discovered during material testing could have a strong influence on the FBG performance and functionality: First, the space that the optical fiber has in the adhesive bond line and the resulting thickness of the adhesive layer between fiber surface and adherent. The stress gradient caused by residual strain and mechanical loading is highest at the interface between the adherent and adhesive layer and decreases towards the center of the adhesive layer [12,13]. When an optical fiber with an FBG completely or almost completely fills the bond line, the FBG will experience a stress gradient, which is likely to cause birefringence effects in the FBG signal. This affects the functionality of the sensor. Sarfaraz et al. [13] investigated the influence of curing temperatures and water uptake on FBG sensor signals and found that FBGs in optical glass fibers with a greater diameter are more strongly affected than FBGs in smaller optical glass fibers. They related this finding to the greater distance between fiber surface and adherent. The results from the photomicrographs of the cross-sectional cuts indicate that, in combination with the tested film adhesives, FBGs in A80 and P125 fibers are at a high risk of experiencing birefringence effects. The risk is likely to be lower for FBGs in P50 and P80 fibers. The same applies for fiber bending caused by the presence of the fabric carrier.

Air inclusions in the adhesive layer are also likely to affect the FBG signal, because they present inhomogeneity in the host material. Thus, the air inclusions might increase the stress distribution in their vicinity [28]. If the air inclusions are located at the interface between optical fiber and adhesive, it is likely that the general stress information from the adhesive host material will not be transferred correctly to the FBG in the fiber core. Measurement errors could occur without being noticed by the user. Furthermore, air inclusions at the interface could act as an initiation point for (partial) debonding between the optical fiber surface and the adhesive host material [29]. They could also lead to measurement errors or the loss of the FBG signal. Therefore, the amount of air inclusions in the host material should be minimized.

A good interface strength between the glass fiber surface, fiber coating, and host material is crucial for the sensor’s performance and functionality [5,30]. A weakening or destruction of the interface caused by the curing temperatures of the CFRP or adhesive host material affects the FBG functionality to a great extent. This makes acrylate-coated FBGs unsuitable for SHM applications in the film adhesives tested here or similar adhesives. Furthermore, the practice of embedding bare optical glass fibers in high-temperature curing adhesives or CFRPs is questionable. The cross-sectional cuts, where all polyimide-coated optical fibers are intact after sample preparation, and all acrylate-coated fibers with separated coatings are destroyed, demonstrates the vulnerability of a bare glass fiber in the host material.

## 5. Conclusions

The present study indicates that it is possible to embed optical glass fibers in structural film adhesive CFRP bonds without significantly reducing the average quasi-static tensile shear strength. However, only polyimide-coated 50 µm and 80 µm glass fibers have suitable diameters and material properties for embedding without a significant reduction of the average quasi-static tensile shear strength. In addition, 125 µm polyimide-coated glass fibers and acrylate-coated 80 µm glass fibers are not suitable for embedding in the structural film adhesives tested here. The tested structural film adhesive (with a layer thickness after curing of 90 µm) appears to be more sensitive to the embedding of different glass fiber types than the tested repair film adhesive (with a layer thickness after curing of 100 µm). In this study, polyimide-coated 80 µm optical fibers were embedded in structural film adhesives and single lap shear fatigue tests were performed. No significant influence on the tensile shear fatigue strength by the embedding of the polyimide-coated 80 µm optical fiber was detected. The fatigue limit of the fatigue specimen carrying the polyimide-coated 80 µm optical fiber was even slightly higher at lower stress levels than that of the reference specimen. This finding makes the tested P80 optical glass fibers very suitable for SHM applications in the aeronautical and even other industries with similar requirements and standards.

## Figures and Tables

**Table 1:** Comparison of the characteristics of the acrylate and polyimide coatings (adapted from [17,18,19]).

Characteristics	Acrylate	Polyimide
Young’s modulus	100 MPa	3 GPa
Maximum operating temperature	85 °C	300 °C
Bonding to fiber surface	Deposited only	Chemically bonded

**Table 2:** Glass fiber types employed in this study. Different glass fiber diameters in combination with either the polyimide or acrylate coating led to the total fiber diameters listed below.

Glass Fiber Type	P50	P80	P125	A80
Cladding diameter	50 µm	80 µm	125 µm	80 µm
Coating material	Polyimide	Polyimide	Polyimide	Acrylate
Total diameter	54–59 µm	95 µm	140 µm	145 µm

**Table 3:** Average quasi-static tensile shear strength and standard deviation of the untreated and aged (water immersion, 70 °C, 1000 h) single lap FM^®^ 300-2K and FM^®^ 300K samples (n = 6).

Tensile Shear Strength [MPa]	Reference	P50	P80	P125	A80
FM^®^ 300-2K, untreated	39.6 ± 0.6	35.4 ± 3.5	38.7 ± 1.4	35.7 ± 4.3	37.3 ± 1.6
FM^®^ 300K, untreated	39.9 ± 2.3	39.8 ± 2.4	38.4 ± 2.5	36.1 ± 1.9	35.1 ± 3.7
FM^®^ 300-2K, 1000 h, 70 °C	27.1 ± 2.7	30.2 ± 3.0	29.5 ± 4.0	28.4 ± 3.0	27.4 ± 2.8
Reduction in %, FM^®^ 300-2K	−32%	−15%	−24%	−20%	−27%
FM^®^ 300K, 1000 h, 70 °C	39.0 ± 2.3	36.5 ± 2.3	38.2 ± 2.5	33.3 ± 1.2	32.1 ± 0.9
Reduction in %, FM^®^ 300K	−2%	−8%	−1%	−12%	−11%
